# Transcriptome data of the carrageenophyte *Eucheuma denticulatum*

**DOI:** 10.1016/j.dib.2019.103824

**Published:** 2019-03-12

**Authors:** Roohaida Othman, Afiq Adham Abd Rasib, Mohammad Akhmal Ilias, Suganthi Murthy, Najihah Ismail, Nursyuhaida Mohd Hanafi

**Affiliations:** a Centre for Biotechnology and Functional Food, Faculty of Science and Technology, Universiti Kebangsaan Malaysia, 43600 UKM Bangi, Selangor, Malaysia; b Institute of Systems Biology, Universiti Kebangsaan Malaysia, 43600 UKM Bangi, Selangor, Malaysia; c Agro-Biotechnology Institute Malaysia, National Institutes of Biotechnology Malaysia, c/o MARDI Headquarters, 43400 Serdang, Selangor, Malaysia

## Abstract

*Eucheuma denticulatum* or commonly known as “Spinosum”, is an economically important red alga that naturally grows on coral reefs with moderately strong currents in tropical and sub-tropical areas. This species is the primary source of iota-carrageenan which has high demands in the food, pharmaceutical and manufacturing industries, and as such it has been widely cultivated. The increasing global demand for carrageenan has led to extensive commercial cultivation of carrageenophytes mainly in the tropics. The carrageenophyte seaweeds including *E. denticulatum* are indigenous to Sabah, Malaysia. To enrich the information on the genes involved in carrageenan biosynthesis, RNA sequencing has been performed and transcriptomic dataset has been generated using Illumina HiSeq™ 2000 sequencer. The raw data and transcriptomic data have been deposited in NCBI database with the accession number PRJNA477734. These data will provide valuable resources for functional genomics annotation and investigation of mechanisms underlying the regulations of genes in this algal species.

**Table:** Specifications table

Subject area	Biology
More specific subject area	Transcriptomics
Type of data	Transcriptome sequences
How data was acquired	Illumina HiSeq™ 2000
Data format	Raw sequences (FASTQ) and processed data (FASTA)
Experimental factors	Thalli of seaweed growing in its natural site
Experimental features	Apical segments of *Eucheuma denticulatum* thalli were used. RNA was extracted from three biological replicates.
Data source location	Semporna, Sabah, Malaysia (4.4794° N, 118.6115° E)
Data accessibility	All data can be accessed at the following link https://www.ncbi.nlm.nih.gov/bioproject/PRJNA477734 GenBank accession for the raw data is accession number SRR7418775 at https://www.ncbi.nlm.nih.gov/sra/SRR7418775/
Related research article	Z.A. Mohamed-Hussein, K.K. Loke, R.A. Zainal-Abidin, R. Othman, EuDBase: An online resource for automated EST analysis pipeline (ESTFrontier) and database for red seaweed *Eucheuma denticulatum*, Bioinformation 7 (4) (2011) 157-162

**Table:** 

**Value of the data** • Current transcriptome datasets improve transcriptomic database of *E. denticulatum.* • The data will add to the transcriptomic resources of carrageenan-producing seaweeds in understanding the molecular regulation of carrageenan biosynthesis. • The data can be useful for comparative analysis with other seaweeds that produce different types of carrageenan.

## 1 Data

Transcriptome of wild type *E. denticulatum* was generated from RNA extracts prepared from apical segments of the thalli. The short reads were filtered, processed, assembled and analyzed as described in the following section. The raw data were deposited on the SRA database with accession number SRR7418775 at https://www.ncbi.nlm.nih.gov/sra/SRR7418775/. The data enhance existing transcriptome database of *E. denticulatum*, EuDBase, which contains expressed sequence tags and transcripts of this seaweed [1].

## 2 Experimental design, materials, and methods

### 2.1 Plant materials

Thalli of *E. denticulatum* were collected from Semporna (4.4794° N, 118.6115° E) in Sabah, Malaysia at 28 °C in the field and washed with sterilized water three times to clean them from any attached contaminants, and subsequently dried on hygroscopic filter paper. The apical segments of the seaweed were frozen in liquid nitrogen and then stored at −80 °C until use.

### 2.2 Total RNA extraction and transcriptome sequencing

Total RNA was extracted from three biological replicates of the seaweed sample using a modified Lopez-Gomez and Gomez-Lim method [2]. Quantity and quality of the total RNA were determined using Nanodrop (Thermo Fisher Scientific Inc., USA) and Agilent 2100 Bioanalyzer (Agilent Technologies, USA), respectively. All three samples of the biological replicates had RNA integrity number (RIN) of around 7.0 or higher and were used for library preparation and sequencing. cDNA libraries construction and sequencing were performed by Macrogen, Inc. (Seoul, Republic of Korea) on Illumina HiSeq 2000 platform (San Diego, USA) according to the manufacturer's instructions. The data from the three libraries were combined together in the assembled transcriptome.

### 2.3 Raw reads processing and assembly

The quality of raw reads was checked using FastQC version 0.11.3 [3]. A total of 44.48 million good quality reads were obtained after the removal of adapter sequences and low quality reads using Trimmomatic version 0.32 with the phred quality cutoff score of 33 [4]. Trinity (r20140717) was then used to perform *de novo* assembly with default parameters which include alignment of the reads to the assembled transcriptome using Bowtie 2 [5]. Statistics of the assembly is as shown in Table 1. The assembled data were deposited into the NCBI database with accession number GGYH00000000. The assembled unigenes were compared with the Non-redundant protein database (NR) (http://www.ncbi.nlm.nih.gov) and UniProtKB (https://www.uniprot.org/help/uniprotkb) using the BLASTX program with the E value ≤ 1e-5. Gene Ontology (GO) (http://www.geneontology.org) annotation was carried out with the Blast2GO program [6] with the E value ≤ 1e-5 and the WEGO tool [7] was used to assign GO classifications. Summary of the GO analysis results are shown in Table 2.

**Table 1:** Statistics of *Eucheuma denticulatum* transcriptome assembly.

Number of assembled bases	4,492,191,140
Number of reads	44,477,140
Total length of transcripts	27,547,155
Total number of transcripts	26,822
Percent GC	52.86%
Contig N90	360 bp
Contig N80	650 bp
Contig N70	1057 bp
Contig N60	1608 bp
Contig N50	2176 bp
Max contig length	16,671
Average contig length	1023
Min contig length	201

**Table 2:** Gene ontology classification of the *Eucheuma denticulatum* transcripts.

GO Terms	Number of Hits
Biological Process	3,603
Cellular Component	3,286
Molecular Function	3,942
No hits	1,5991
