# Supplementary material for: Transcriptome data of the carrageenophyte Eucheuma denticulatum
Source: Data Brief. 2019 Mar 12;24:103824. doi: 10.1016/j.dib.2019.103824 (PMC6444127; doi:10.1016/j.dib.2019.103824)
Supplement: Multimedia component 1 [file mmc1.docx]

**Conflict of interest**

All the authors have approved submission and there are no conflicts of interest.
